# Middle aortic syndrome presenting with megacolon

**DOI:** 10.1093/ehjcr/ytad536

**Published:** 2023-10-27

**Authors:** Takao Konishi, Mikoto Yoshida, Susumu Matsukuma, Akimi Uehata

**Affiliations:** Department of Cardiovascular Medicine, Faculty of Medicine and Graduate School of Medicine, Hokkaido University, West 7, North 15, Kita-ku, 060-8638 Sapporo, Japan; Department of Cardiology, Gunma Chuo Hospital, Maebashi, Japan; Department of Pathology and Laboratory Medicine, National Defense Medical College, Tokorozawa, Japan; Division of Cardiology, Kisen Hospital, Tokyo, Japan

A 72-year-old man, who had a history of treatment-resistant hypertension due to aortic stenosis and underwent subclavian-iliac bypass surgery 20 years before, visited the emergency department due to abdominal pain and distention. Abdominal X-ray and sagittal computed tomography showed prominent dilatation of the ascending colon (*Panels A* and *B*, asterisks). Computed tomography angiography revealed a localized cross-sectional luminal narrowing in the abdominal aorta (*Panel C* and arrow in Panel D), compared to the lumen of the more proximal descending aorta (*Panel C* and arrowhead in Panel E). A bypass graft to the iliac artery (*Panel C*, arrow) and numerous collateral arteries (*Panel C*, arrowheads) were also visualized. Although his symptoms were improved after the placement of transanal ileus tube, he died of sepsis due to gastrointestinal and urinary tract infection on Day 20 after admission. Autopsy revealed the severe narrowing of the abdominal aortic lumen at the level of superior mesenteric artery (SMA) (*Panel F*). Histological examination demonstrated marked fibrous thickening of the aortic intima resulting in the abdominal aortic stenosis and occlusion of SMA (*Panel G*, arrowheads). The cross-sectional area luminal narrowing of the most stenotic abdominal aorta was calculated as 94.8% (*Panel H*). Middle aortic syndrome (MAS) is a rare vascular disease characterized by the hypoplasia or coarctation of distal descending thoracic aorta and/or abdominal aorta. Although typical symptoms of MAS include severe hypertension, headache associated with high blood pressure, bilateral lower-limb claudication, and renal failure, megacolon as a main symptom of MAS is extremely rare. Clinicians should recognize MAS as a possible cause of megacolon.

**Figure:**
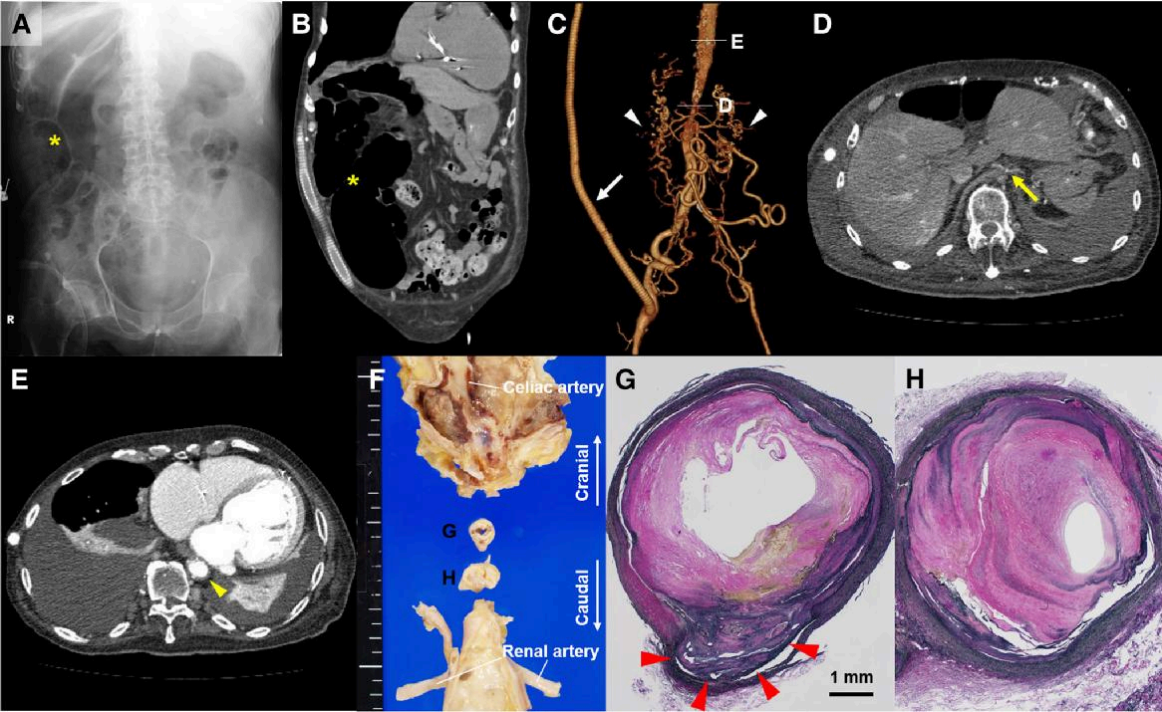


X-ray, computed tomography, and pathology in a case of middle aortic syndrome presenting with megacolon. (*Panels A* and *B*): (*Panel A*) Abdominal X-ray and (*Panel B*) computed tomography showing megacolon. (*Panels C–E*) Computed tomography angiography showing a localized luminal narrowing in the abdominal aorta. (*Panels F–H*) (*Panel F*) Macroscopic and (*Panels G* and *H*) microscopic images showing a severe stenosis in the abdominal aorta and a total occlusion of the superior mesenteric artery.

## Data Availability

The data included in this article will be shared upon reasonable request to the corresponding author.

## Notes

**Consent:** The patient reported in this case is deceased. The authors have been unable to contact the patient’s next-of-kin to obtain consent for publication, but every effort has been made to anonymize the case. This situation has been discussed with the editors.

**Funding:** This work was supported in part by The Ito Foundation (The 27th Ito Foundation Grant, TK).

